# Clade homogeneity and *Pol* gene polymorphisms in chronically HIV-1 infected antiretroviral treatment naive patients after the roll out of ART in Ethiopia

**DOI:** 10.1186/1471-2334-14-158

**Published:** 2014-03-22

**Authors:** Andargachew Mulu, Thomas Lange, Uwe Gerd Liebert, Melanie Maier

**Affiliations:** 1 Institute of Virology, Leipzig University Leipzig, Germany; 2 Department of Microbiology, College of Medicine and Health Sciences, University of Gondar Gondar, Ethiopia

**Keywords:** Antiretroviral drug resistance, HIV subtype, Protease, Reveres transcriptase

## Abstract

**Background:**

Despite the increasing use of antiretroviral treatment (ART) recent data on frequency and pattern of drug resistance mutations in Ethiopia is not available. Furthermore with increasing mobility of people HIV-1 subtypes other than the predominant subtype C may likely be introduced from the neighbouring countries. This study was aimed to determine the molecular characterization and pre-antiretroviral treatment resistance mutations among HIV-1 chronically infected ART naïve patients after the roll out of ART in Ethiopia.

**Methods:**

Viral RNA was determined in 160 baseline plasma samples. The entire PR and the first 335 codons (76%) of the RT regions of the *pol* gene of the HIV-1 genome (N = 160) were amplified and sequenced using an in-house assay. Genotypic drug resistance was defined as the presence of one or more resistance-related mutations as specified by the consensus mutation of Stanford University HIVDB and the International Antiviral Society (IAS) mutation lists.

**Results:**

A predominance of HIV-1 subtype C (98.7%) was observed. The level of drug resistance is found to be 5.6% and 13.1% according to the Stanford University HIVDB drug resistance interpretation algorithms and the International Antiviral Society mutation lists, respectively. Mutations conferring simultaneous resistance to NRTIs and NNRTIs were not detected and no major PR mutation was found. However, a high rate of polymorphic changes both in PR and RT regions were observed. Moreover, twenty four (15%) monophyletic transmission clusters with bootstrap value of 99% were found.

**Conclusions:**

Strong evidence for consistent HIV-1C clade homogeneity and low influx of other variant into the country was found. The level of drug resistance observed in chronically infected treatment naïve patients which exceeds the WHO estimates suggests the need for incorporation of HIV-1 drug resistance testing prior to ART initiation. The occurrence of monophyletic transmission clusters affecting (24/160) individuals indicates their potential risk related practice. Thus, an intensified public health intervention program and monitoring of HIV drug resistance testing appears indispensible.

## Background

In response to the global HIV-1 epidemic and to achieve universal access for the treatment of HIV-1 infected individuals in resource limited countries, antiretroviral treatment (ART) has been scaled up in the past decade [1]. However, the emergence of drug resistant variants [2] and their transmission to newly infected individuals has been documented [3]. Studies from industrialized countries have shown a decline or a stabilized incidence in transmitted drug resistance (TDRs) with time related to nonucleoside reverse-transcriptase inhibitors (NNRTIs) [4,5]. On contrary, available data from African countries showed an increasing trend on the prevalence of TDR over time after the roll out of ART in the region [4-6].

In Ethiopia, TDR before the introduction of ART (2002) and one year after its introduction (2005) was reported well below 3% [7,8]. Nevertheless, data on drug resistance mutation after the scale up of ART are not available. With the increasing availability of ART in Ethiopia in recent years, the prevalence of TDRs will presumably also increase. Previous studies have shown that, HIV-1C is predominantly circulating in 99% of the infected individuals in Ethiopia unlike other sub-Saharan-Eastern African countries [9,10]. However, with increasing mobility and migration of people, HIV-1 variants may be introduced and intermixed from the neighbouring countries where subtypes A, D, G as well as recombinant forms in Kenya and subtype D in Sudan are co-circulating [9,10].

HIV pol gene encodes the reverse-transcriptase (RT), protease (PR) and integrase enzymes that are the major targets of antiretroviral therapy [11]. As drug-regimens used in Southern and Eastern African countries, where non-B subtypes are predominately circulating mostly consist of NNRTIs and NRTIs in the 1^st^ with addition of protease inhibitors (PIs) in 2^nd^ line, understanding of drug-resistance patterns in *pol* gene among non-B subtypes may help to optimize the selection of first-line regimens and limit the acquisition of cross-resistance. The objectives of the current study is to determine the HIV-1 genetic diversity and to identify the pattern of antiretroviral drug resistance mutations in *pol* gene of HIV-1 isolated from chronically infected treatment naïve Ethiopian patients.

## Methods

### Patients

HIV-1 chronically infected treatment naïve patients (N = 160) with advanced diseases (WHO clinical stages III and IV) [12] above 18 years of age and seeking care and treatment at Gondar University Hospital for the first time, Northwest Ethiopia in 2008/2009 were recruited consecutively. Patients were excluded for the following reasons: pregnant or had taken single dose nevirapine (NVP) for prevention of mother to child transmission (PMTCT) or patients with known chronic illness or any previous ART use.

### Blood collection

Five ml venous blood was collected in vacutainer tubes containing ethylene diamine tetraacetic acid (EDTA). Baseline CD4^+^ T cell count was measured using the FACSCount flow cytometer (Becton Dickinson, San Jose, CA, USA) following the manufacturer’s protocol. Plasma was separated by centrifugation and stored at −40°C.

### RNA extraction and plasma viral load determination

RNA extraction was done with the Abbott m2000sp automated sample preparation system using mSample preparation system RNA kit. Plasma viral load was determined with Abbott m2000rt Quantitative RealTime HIV-1 assay (Abbott Molecular, Des Plaines, IL, USA) with a lower detection limit of 40 copies/ml.

### Reverse transcription and PCR amplification for *pol* gene sequencing

The entire PR and the first 335 codons (76%) of the RT regions of the pol gene of the HIV-1 genome of 160 patients were amplified with an in-house protocol as described before [13]. Briefly, RNA elute was reverse transcribed using AMV reverse transcriptase (Promega Corporation, WI, USA) by an outer primer HIVrt (Table 1). Viral cDNA was amplified by nested PCR using Phusion Hot Start High-Fidelity DNA polymerase (Finnzymes, Espoo, Finland) by outer primers HIVpcrFor1 and HIVpcrRev1 (yielding a 1757 bp amplicon) and subsequently by the inner primers HIVpcrFor2 and HIVpcrRev2 (yielding a 1389 bp amplicon, Table 1). Initial denaturation was done at 98°C for 2 min followed by 40 cycles consisting of 10 sec of denaturation at 98°C and 25 sec of annealing at 64°C for the first round and at 53°C for the second round with a 40 sec extension at 72°C for both and final extension for 5 min at 72°C.

**Table 1:** **List of in-house primers used for *pol *** **genome amplification and genotypic drug-resistance testing**

**Name**	**Sequence**	Position*	**Use**
HIVrt	5’TGTTTTACATCATTAGTGTG 3’	3630–3649	Pol/outer
HIVpcrFor1	5’TGATGACAGCATGTCAGGGAGTGG 3’	1826–1849	Pol/outer
HIVpcrRev1	5’GGCTCTTGATAAATTTGATATGTCCATTG3’	3555–3583	Pol/outer
HIVpcrFor2	5’AGCCAACAGCCCCACCAG 3’	2150–2167	Pol/inner
HIVpcrRev2	5’CTGTATTTCTGCTATTAAGTCTTTTG 3’	3514–3539	Pol/inner
HIVseq1	5’GTTAAACAATGGCCATTGACAGA 3’	2610–2632	Pol/inner
HIVseq2	5’TGGAAAGGATCACCAGCAATATT 3’	3006–3028	Pol/inner
HIVseq3	5’GGGCCATCCATTCCTGGCT 3’	2584–2605	Pol/inner
HIVseq4	5’CCATCCCTGTGGAAGCACATT 3’	2988–3008	Pol/inner

*All positions are matched to HIV-1HXB2 (GenBank Accession number K03455).

### Sequencing

Purified PCR products were subjected to direct sequencing of both sense and antisense strands using Big Dye Terminator Cycle Sequencing Ready Reaction kit (Applied Biosystems, Foster City, CA, USA). For each sample, six separate sequencing reactions were done using the two inner PCR primers (HIVpcrFor2 and HIVpcrRev2) and four additional internal primers: HIVseq1, HIVseq2, HIVseq3 and HIVseq4 (Table 1) which allowed a double coverage of the *pol* region. All primer positions are matched to HIV-1HXB2 (GenBank accession number K03455). Both forward and reverse overlapping sequences were manually edited with the Geneious software version 5.4 [14].

### Phylogenetic analysis

*Pol* gene sequences were aligned with reference subtypes (A-D, F-H, J, K, circulating recombinant forms (CRFs) and SIV) obtained from HIV Sequence Database at Los Alamos (http://www.hiv.lanl.gov) accessed on September 24, 2013. Phylogenetic inferences were performed by the neighbour-joining method with 1,000 bootstrap replicates under Kimura’s two-parameter correction using MEGA 5. The evolutionary distances were computed using the Maximum Composite Likelihood method and are in the units of the number of base substitutions per site [15].

### Drug resistance analysis

The existing drug resistance interpretation algorithms use lists of amino acid mutations that are associated with drug resistance, derived largely from research of HIV-1B infected individuals [16-20]. With this limitation, in this study genotypic drug resistance was defined as the presence of one or more resistance-related mutations, as specified by the consensus mutation of Stanford University HIVDB (http://hivdb.stanford.edu) accessed on September 24, 2013 and the latest definition of the International Antiviral Society (IAS) mutation lists [16].

### Statistical analysis

Age, sex, WHO clinical stage, CD4^+^ T cell count and HIV RNA level were included in the statistical analysis using SPSS statistical software (Version 17, USA). Student *t* test was used and a p-value of < 0.05 was considered statistically significant.

### Ethical issue

Ethical clearance and written informed consent from the University of Gondar Ethical Review Committee (RPO/55/291/00) and each study subjects was obtained, respectively.

## Results

### Patient’s characteristics

One hundred and sixty samples (73 males and 87 females) were amplified and sequenced for HIV-1 subtyping and genotypic drug resistance analysis. The mean ± SD age of the subjects was 32.3 ± 8.5 years (range 18–66 years). The socio-demographic, clinical, immunological and virological characteristics of the patients are summarized in Table 2. The mean log_10_ HIV RNA level of the patients was significantly lower (4.46log_10_ versus 5.51log_10_) in patients with higher (>200 cells/mm^3^) compared with lower (<200 cells/mm^3^) CD4^+^ T cell strata (P < 0.001).

**Table 2:** Sociodemographic, immunological and virological profile of chronically HIV-1 infected patients from Northwest Ethiopia

**Variables**	**Male no. (%)**	**Female no. (%)**	**Total no. (%)**
	**73 (45.4)**	**87 (54.1)**	**160 (100.0)**
Age (years)			
<20	1 (1.4)	2 (2.3)	3 (1.9)
20–29	19 (26.0)	45 (51.7)	64 (40.0)
30–39	33 (45.2)	24 (27.6)	57 (35.6)
40–49	15 (20.6)	12 (13.8)	27 (16.9)
≥50	5 (6.8)	4 (4.6)	9 (5.6)
WHO clinical status			
I	6 (8.2)	13 (14.9)	19 (11.9)
II	13 (17.8)	15 (17.2)	28 (17.5)
III	49 (67.1)	57 (65.5)	106 (66.3)
IV	5 (6.8)	2 (2.3)	7 (4.4)
CD4^+^ T cell count (cells/mm^3^)			
<100	26 (35.6)	26 (29.9)	52 (32.5)
100–200	16 (21.9)	31 (35.6)	47 (29.4)
201–350	29 (39.7)	29 (33.3)	58 (36.3)
>350	2 (2.7)	1 (1.1)	3 (1.9)
HIV RNA level (copies/ml)			
10^3^–10^4^	13 (17.8)	16 (18.4)	29 (18.1)
10^4^–10^5^	28 (38.4)	38 (43.7)	66 (41.3)
10^5^–10^6^	29 (39.7)	28 (32.2)	57 (35.6)
>10^6^	3 (4.1)	5 (5.7)	8 (5.0)

### HIV-1 subtypes

**Figure 1:**
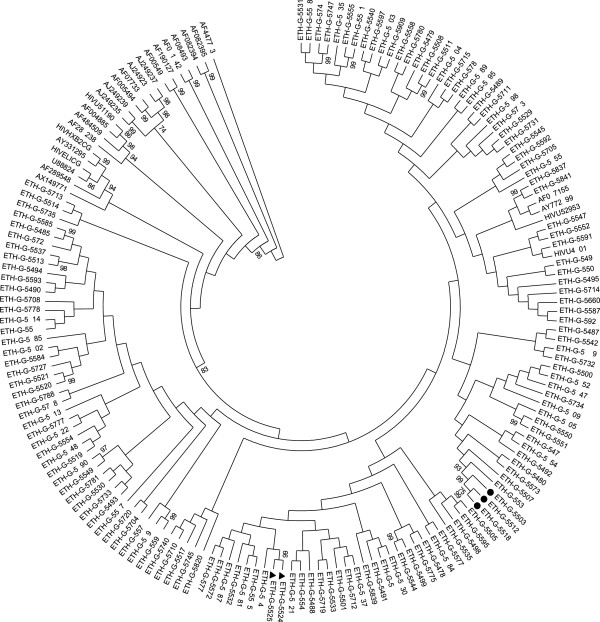
**Phylogenetic tree of the HIV-1 *pol *regions at nucleotide level aligned with reference sequences from the Los Alamos database (http://www.hiv.lanl.gov).** Bootstraps values (1000 replicates) >70% are shown. Current isolates are designated as ETH-G-followed by 4 digit number and the rest are reference sequences. Keys: ●: Confirmed monophyletic groups come from one Muslim man who had married with two women. ►: Confirmed monophyletic groups come from another heterosexual couple. Reference sequences: HIVU46016 (C-Ethiopia), AY772699 (C-S. Africa), AF067155 (CIndia), HIVU52953 (C-Brazil), AX149771 (BC-China) and the rest references sequences are non C.

All sequences clustered with HIV-1C in HIV pol genome with a bootstrap value of 83% (Figure 1). However, 2 patients had discordant HIV-1 subtypes according to HIVDB: In one sample (ETH-G-5768) PR and RT was identified as HIV-1 subtype D and C, respectively and in another (ETH-G-5685) PR and RT was sub-typed as subtype K and C, respectively. Twenty four (15%) closely related monophyletic transmission clusters with bootstrap value of 99% were observed (Figure 1). Of which samples ETH-G-5518, ETH-G-5512 and ETH-G-5503 are confirmed samples come from heterosexual couples in which one Muslim man had married with two women; ETH-G-5524 and ETH-G-5525 derived from another confirmed heterosexual couples. Definite phylogenetic relation with a particular subtype C isolates from other countries where subtype C circulates (S. Africa, India and Brazil) was also observed (Figure 1).

### Genotypic drug resistance

**Figure 2:**
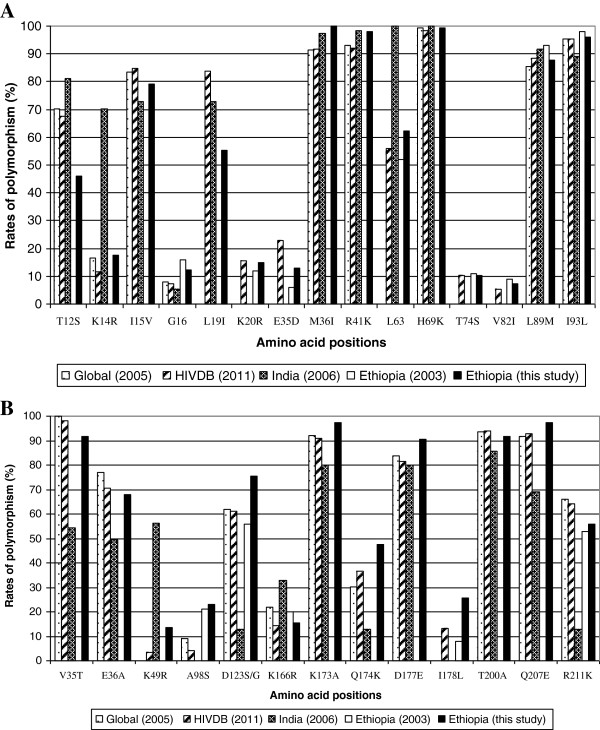
**Rate of polymorphism at the PR (A) and RT (B) region of the HIV-1 *pol *** **gene among chronically infected treatment naïve patients in Ethiopia compared with the universally established subtype C specific polymorphisms and other subtype C isolates.**

According to the IAS and the HIVDB interpretation algorithms 13.1% (21/160) and 5.6% (9/160), respectively were found to have one or more mutation conferring resistance to RTIs (Table 3). There was no major drug resistance mutation in PR region. In both algorithms, simultaneous resistance to NRTIs and NNRTIs (Table 3) was not detected. In addition, minor mutations that might contribute to PIs resistance and a number of polymorphic changes have been observed in both PR (Table 4, Figure 2A) and RT regions (Table 4, Figure 2B).

**Table 3:** Resistance conferring mutations in the RT gene among chronically infected patients from Northwest Ethiopia

			**NRTI**		**NNRTI**	
**Lab no**	**Age/Sex**	HIV load*	***Mutation***	***Resistance to***	***Mutation***	***Resistance to***
5479	25 yrs/f	5.44	-	-	*E138G*	ETR
5480	35 yrs/f	4.20	-	-	**Y188H**	ETR, DLV, EFV, NVP
5905	35 yrs/f	4.58	**K219E**	AZT, D4T, ABC, DDI, TDF	-	-
5496	30 yrs/f	3.88	**L210W**	AZT, D4T	-	-
5489	28 yrs/f	5.76	-	-	*E138G*	ETR
5520	35 yrs/f	5.49	-	-	*E138A*	ETR
5616	30 yrs/f	4.31	**K65R**	ABC, ddI, FTC, 3TC, d4T, TDF	-	-
5652	40 yrs/f	5.50	-	-	*E138A*	ETR
5763	45 yrs/f	4.91	-	-	*E138A*	ETR
5843	27 yrs/f	4.49	-	-	*E138A*	ETR
5501	27 yrs/f	4.38	-	-	*V90I*	ETR
5604	44 yrs/f	4.64	-	-	*V90I*	ETR
5491	25 yrs/m	5.94	-	-	**K101E**, *E138A*	DLV, EFV, ETR, NVP
5533	32 yrs/m	5.17	-	-	**G190A**	EFV, ETR, NVP, RPV
5566	30 yrs/m	5.49	-	-	*E138A*	ETR
5711	22 yrs/m	5.13	-	-	*E138A*	ETR
5727	35 yrs/m	4.80	-	-	*E138A*	ETR
5712	28 yrs/m	4.82	-	-	**G190A**	EFV, ETR, NVP, RPV
5710	38 yrs/m	5.17	**L210W**	AZT, D4T	-	-
5603	22 yrs/m	5.12	-	-		V90I ETR
5991	24 yrs/m	4.36	-	-	**M230I**	RPV

*HIV load in log_10_ copies/ml; Mutations in bold are only considered by both IAS and Stanford University HIV drug resistance algorithm; Mutation in *Italics* are reported by IAS only.

*Abreviations*: *3TC* lamiduvine, *ddI* didanosine, *d4T* stavudine, *FTC* emtricitabine, *TDF* tenofovir, *ZDV* zidovudine, *NNRTI* non-nucleoside RT inhibitors, *DLV* delavirdine, *EFV* efavirenz, *ETR* etravirine, *NVP* nevirapine, *RPV* rilpivirine.

**Table 4:** Polymorphisms at known drug resistance positions of HIV-1 subtype B among chronically infected patients from Northwest Ethiopia (N = 160)

**Drug class**	**Polymorphisms**	**Number of cases**	**Frequency (%)**
***NRTI***		16	10.0
	M41R/N	2/1	1.9
	D67E/G/K	1/1/1	1.9
	Q151P/Q/R	1/1	1.3
	M184L/M	4	2.5
	T215A/P/T	3/1	2.5
***NNRTI***		52	33.5
	A98S/Q	38/1	24.4
	L100V	1	0.6
	K101R/X	3/1	2.5
	K103E/R	2	1.3
	V108T	1	0.6
	E138Y/D	1/1	1.3
	G190 D/R	1/1	1.3
***PR***		11	6.9
	V32A/V	1	0.6
	L10I	3	1.9
	L10V	2	1.3
	L10L/R	1	0.6
	V11I/V	1	0.6
	V11F/V	1	0.6
	V11G/V	1	0.6
	F53L	1	0.6

Keys: Numbers correspond to amino acid positions. The first letter corresponds to the wild-type amino acid; the substituted amino acid is coded by the last letter.

Five patients had NNRTI resistance (K101E in one, Y188H in one, G190A in two and M230I in one patient) according to the Stanford University drug resistance database and IAS mutation lists (Table 3). In addition, according to IAS (Table 3) etravirine resistance associated mutations V90I and E138A/G were observed in 3 (1.9%) and 10 (6.5%) of the patients, respectively which increase the overall magnitude of NNRTI resistance to 10.6% (17/160). One patient had both K101E (in both IAS and/or Stanford algorithms) and E138A mutations (in IAS algorithm).

As indicated in Table 4, a polymorphic mutation K101R was observed in another patient. The polymorphic mutation K103R which occurs in 1-2% of untreated persons and reduces NVP, DLV and EFV susceptibility was also observed in one patient. In addition, highly unusual mutations at known subtype B drug resistance position conferring resistance to NNRTI were observed (Table 4). In 23.7% (38/160) of the patients a polymorphic change from A to S at codon 98 was observed, a substitution that was recently introduced into the ANRS algorithm as conferring resistance to nevirapine in subtype C only (http://www.hivfrenchresistance.org).

In both algorithms, four patients (2.6%) had NRTI resistance-associated mutations. K65R was observed in one patient. Mutations at position 210 from L to W and at 219 from K to E were observed in two and one patient, respectively. A revertant mutation T215A was also observed in 3 (1.9%) of the patients. Moreover atypical mutations at known subtype B drug resistance positions (Table 4) conferring resistance to NRTI were observed at positions M41 (2.3%), D67 (2.3%), Q151 (1.5%), M184 (3.1%) and T215 (2%).

### Drug resistance versus immunological and virological data

Table 3 depicts individual mutation profiles of chronically infected treatment naïve patients and their resistance to distinct antiretroviral drugs. Most resistant viruses were detected in females. There was no significant difference in the plasma HIV RNA level (P value = 0.152) and CD4^+^ T cell counts (P value = 0.170) of patients with and without drug resistance mutations (Table 5). However, among the patients with drug resistance mutation, the majority were observed in patients with the age of greater than 25 years (Tables 3 and 5).

**Table 5:** Characteristics of patients with and without transmitted drug resistance

	**Patients with resistance mutations**	**Patients without resistance mutations**	***P value***
Age (mean ± SEM)	32.7 ± 1.7	32.9 ± 0.78	0.072
Sex			
	Male (number)	8	65
	Female (number)	12	75
CD4^+^ T cells^a^	141 ± 21.5	178 ± 9.3	0.152
Plasma viral load^b^	4.98 ± 0.13	4.73 ± 0.06	0.170

^a^T cell count in numbers/mm^3^.

^b^log_10_ HIV RNA in copies/ml.

## Discussion

This study provides the first description of HIV-1 drug resistance in chronically infected ART naive patients after the scale up of ART in Ethiopia and shows an increase in magnitude of TDR with expanded access of ART in the country and is in line with reports from eastern and southern African countries [4,6]. The results also highlight the need to scale up PIs in the country. The two fold variation in the magnitude of drug resistance between IAS drug resistance mutations lists (13.1%) and HIVDB DR Interpretation algorithm (5.6%) indicates the difficulty of interpreting drug resistance mutation in subtype C isolates [16-20]. The level of drug resistance in the current study is significantly higher than the previous report from the same area (3.3%) sampled in 2002 [7] but similar with findings from other eastern and southern Africa countries after the roll out of ART [4-6] which support the notion that scaling up of ART in Africa will drive the emergence of TDR. The findings also imply that many HIV infected individuals receiving ART are continuing to exercise risk related behaviour which is in line with behavioural study in Ethiopia [21] and an eventual increase of TDR cases is anticipated in the country. Thus, an intensified public health intervention program directed at such patients to prevent TDR viruses is warranted.

In contrast to a previous report among pregnant women from central Ethiopia [8] and other African countries in which TDR was interpreted based on the WHO HIV drug resistance threshold survey, the frequency of resistant associated mutation in the current study is higher than the WHO HIV drug resistance threshold survey report [22] which is similar with a recent study from Tanzania [23]. Nevertheless, it is difficult to directly compare the data from these studies since there were differences in the inclusion criteria, the representativeness of the samples and the resistance algorithms used for interpretation. The current study was a hospital based study among chronically infected patients seeking treatment but the studies for WHO TDR surveillance were based on recently seroconverted individuals below 25 years of age with CD4^+^ T cell counts of >500 cells/ml and primigravida women.

Although the mutations associated with resistance to NNRTIs in subtype C isolates in this study were similar with previous studies from other African countries [4,6] different patterns and frequencies of specific mutations were observed with 10.3% prevalence of drug resistance to this drug classes according to the IAS resistance mutation list. The detection of mutations conferring resistance to NVP (G190A and Y188H) in 4 patients is high compared with previous report from Ethiopia [7,8] and other African countries [4,6] before the scale up ART which may be associated with the prophylactic use of single dose NVP for the PMTCT in the country [22] and the low genetic threshold for resistance to NNRTI. It is also worse enough to detect etravirine associated mutations (V90I, K101E and E138A) in 8.4% of the patients in areas where etravirine is yet not available. This is much higher than the previous reports [7,8] indicating the higher evolutionary rate of HIV-1C in Ethiopia and that HIV-1 variants containing these mutations are circulating in the absence of etravirine in the country. Thus, the introduction of etravirine in African countries where subtype C is predominantly circulating needs attention.

The 2.6% frequency of resistance mutation to NRTIs is low compared with studies from other African countries [4,6]. Low rate of K65R mutation which causes intermediate resistance to most of the NRTIs and low level resistance to d4T [24,25] was observed in one patient and could be due to the recent availability of tenofovir in Ethiopia. Although this mutation is relatively uncommon, there has been an increase in its prevalence in some countries as a result of the widespread clinical use of tenofovir [26-29]. Moreover, despite the wide use of thymidine analogs (zidovidine or stavudine) in Ethiopia, thymidine analog mutations were observed in low frequency unlike previous similar studies [4].

The absence of major drug resistance mutation in PR region is consistent with previous studies [4-7] and might be related to the late introduction and limited access of this drug class in the region. However, the rate of minor mutations (Table 4) is consistent with the previous studies among subtype C isolates [17,30-32]. Although the clinical significance of these natural polymorphisms in non-B subtypes is controversial [33], it is suggested that they may influence the risk of treatment failure, as it does for clade B viruses [34] and may facilitate the selection of different pathways and/or a more rapid emergence of drug resistance and treatment failure [32,35].

The present data clearly shows the predominance (98.7%) of HIV-1 subtype C in Ethiopia similar with previous reports [7-10] and provides a strong evidence for consistent HIV-1C clade homogeneity in the country for the past 4 decades. The reason for the predominance of subtype C in Ethiopia cannot be given with absolute certainty. However, it can be speculated that this subtype when introduced first into the country, has rapidly saturated the commercial sex workers network [9,10,35]. Although, the lack of other subtypes suggest a low influx of subtypes from neighbouring countries [9,10] the rare findings of the inter-subtype recombinant CD and CK may have epidemiological contribution to HIV epidemic in Ethiopia. Moreover, the 15% monophyletic transmission clusters observed in current isolates imply that many HIV infected individuals are exercising risk related behaviour and highlights the importance of understanding transmission dynamics of HIV-1 subtype C in the population.

## Conclusion

In conclusion, there is strong evidence for consistent HIV-1C clade homogeneity and low influx of other HIV-1 subtypes to Ethiopia for the past 4 decades. The observed level of drug resistance which exceeds the WHO’s estimates suggests the need for routine HIV-1 genotypic drug resistance testing for treatment naïve patients and supports the hypothesis that scaling-up of ART in resource limited settings drive the development of drug resistance. The absence of major drug resistance mutations in PR gene suggest for scaling-up of PIs in Ethiopia. The occurrence of monophyletic transmission clusters in 15% of individuals indicate their potential of risk related practice and thus, an intensified public health intervention program targeting such individuals is warranted.

### Sequence data

Nucleotide sequences are deposited in National Centre for Biotechnology Information (NCBI), USA GenBank (Accession Number: KF026059-KF026220).

## Competing interest

We declare no competing interest.

## Authors’ contribution

AM: Conception and design of the study, acquisition, analysis and interpretation of data, drafting the article and final approval of the version to be submitted; TL: Acquisition, analysis and interpretation of data, revising the draft article and final approval of the version to be submitted; UGL: Conception and design of the study, analysis and interpretation of data, revising the draft article and final approval of the version to be submitted; MM: Conception and design of the study, analysis and interpretation of data, revising the draft article and final approval of the version to be submitted.

## Pre-publication history

The pre-publication history for this paper can be accessed here:

http://www.biomedcentral.com/1471-2334/14/158/prepub
